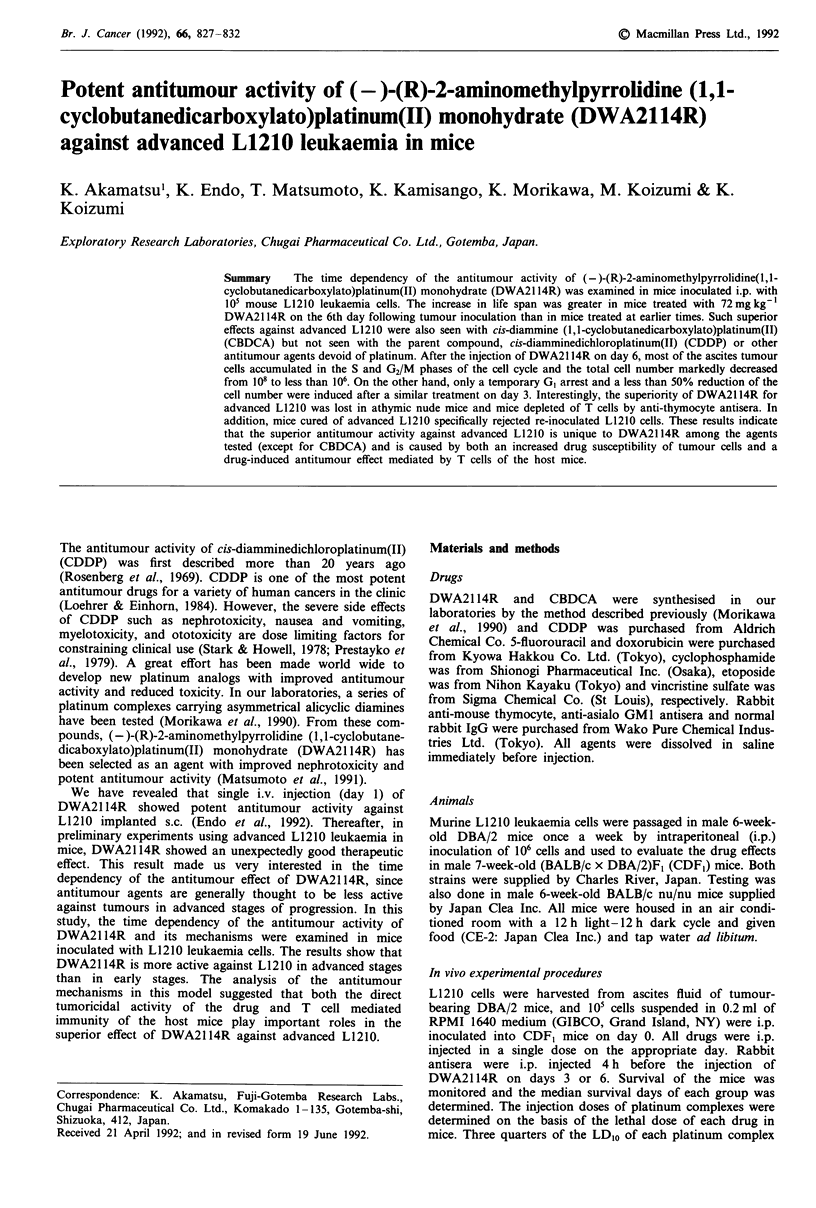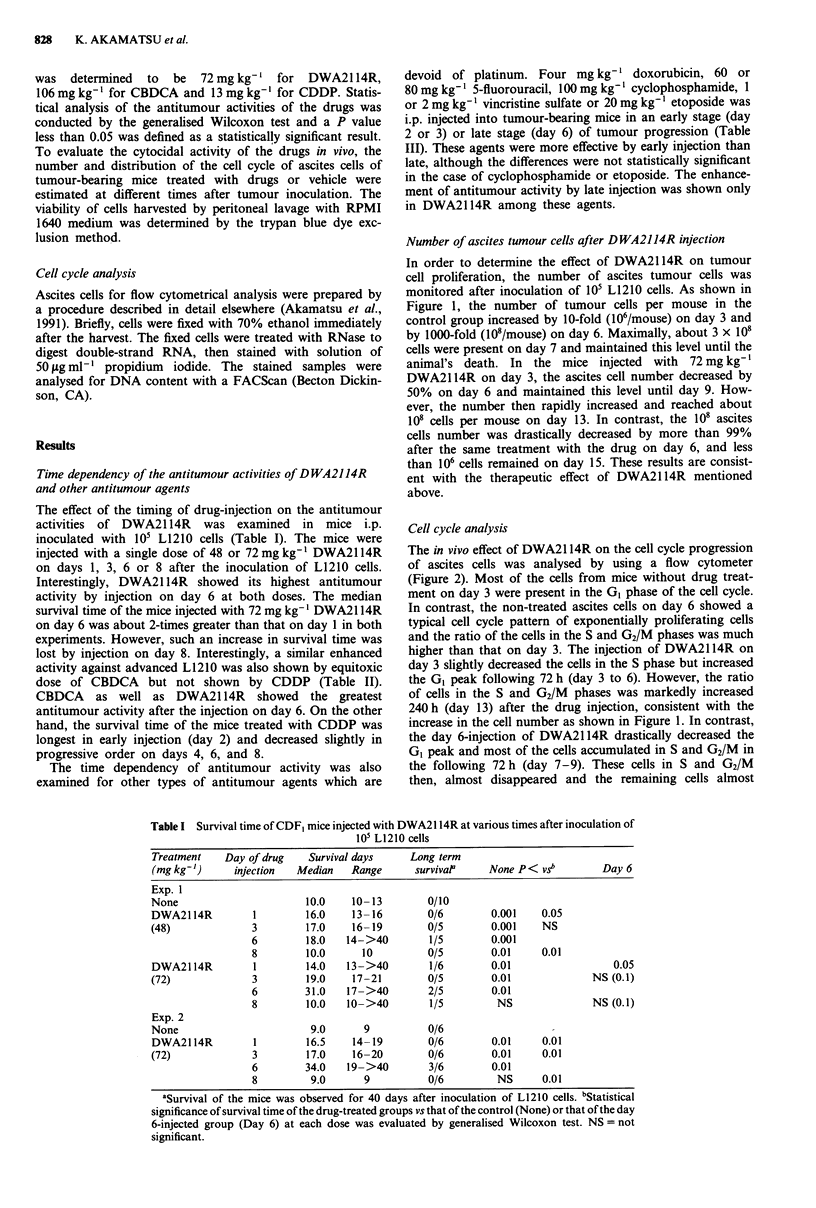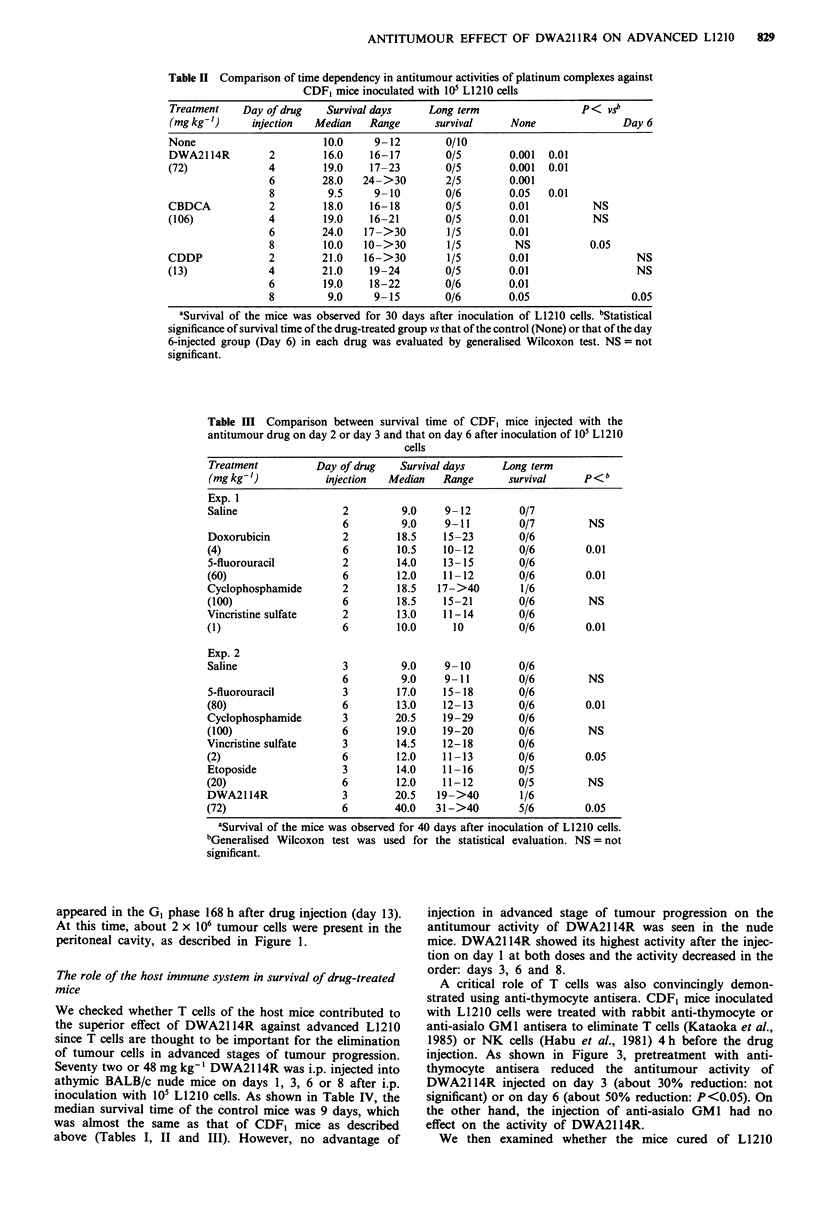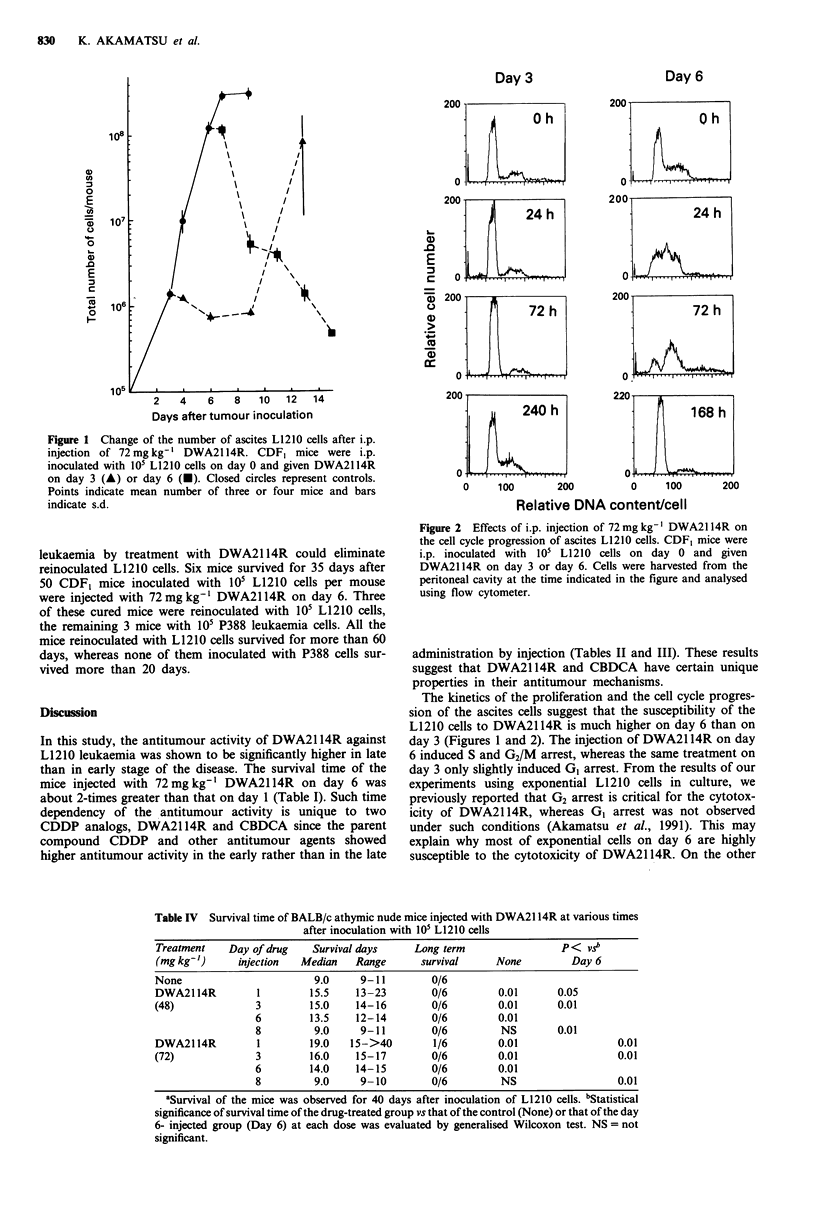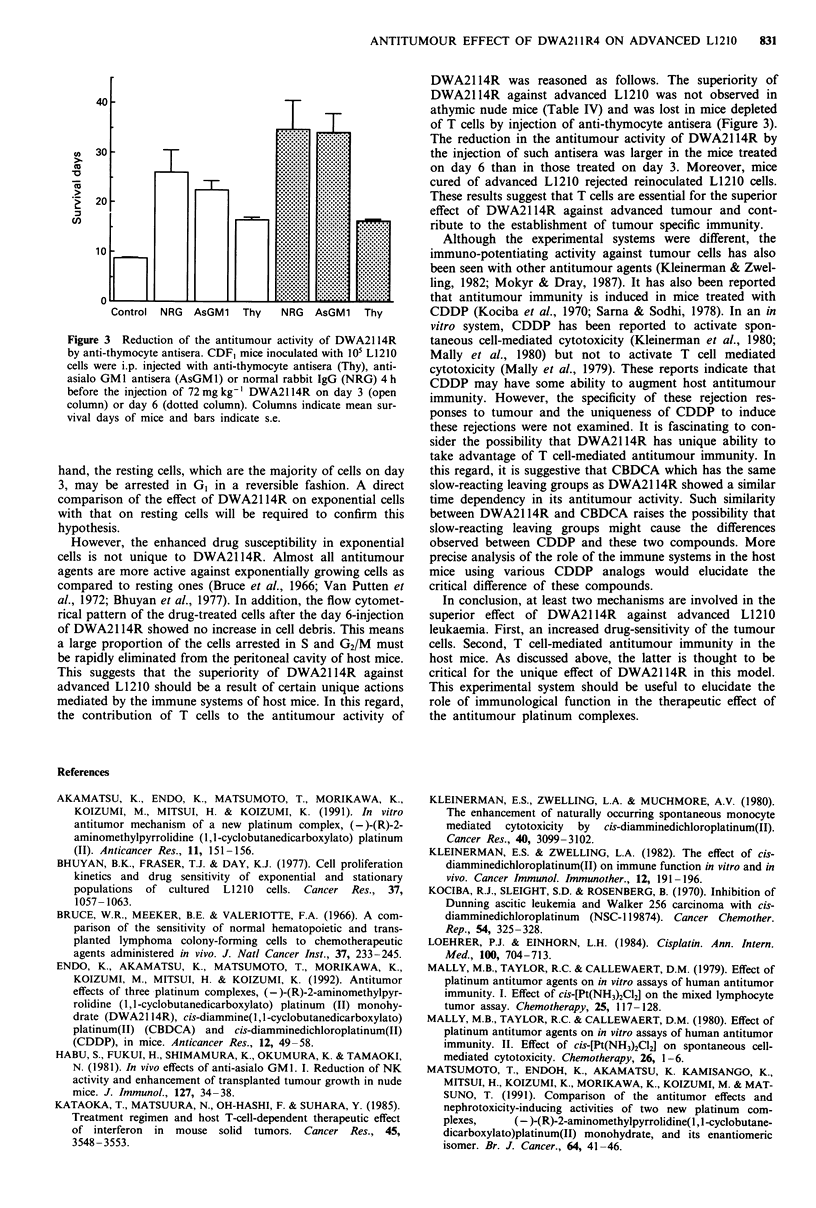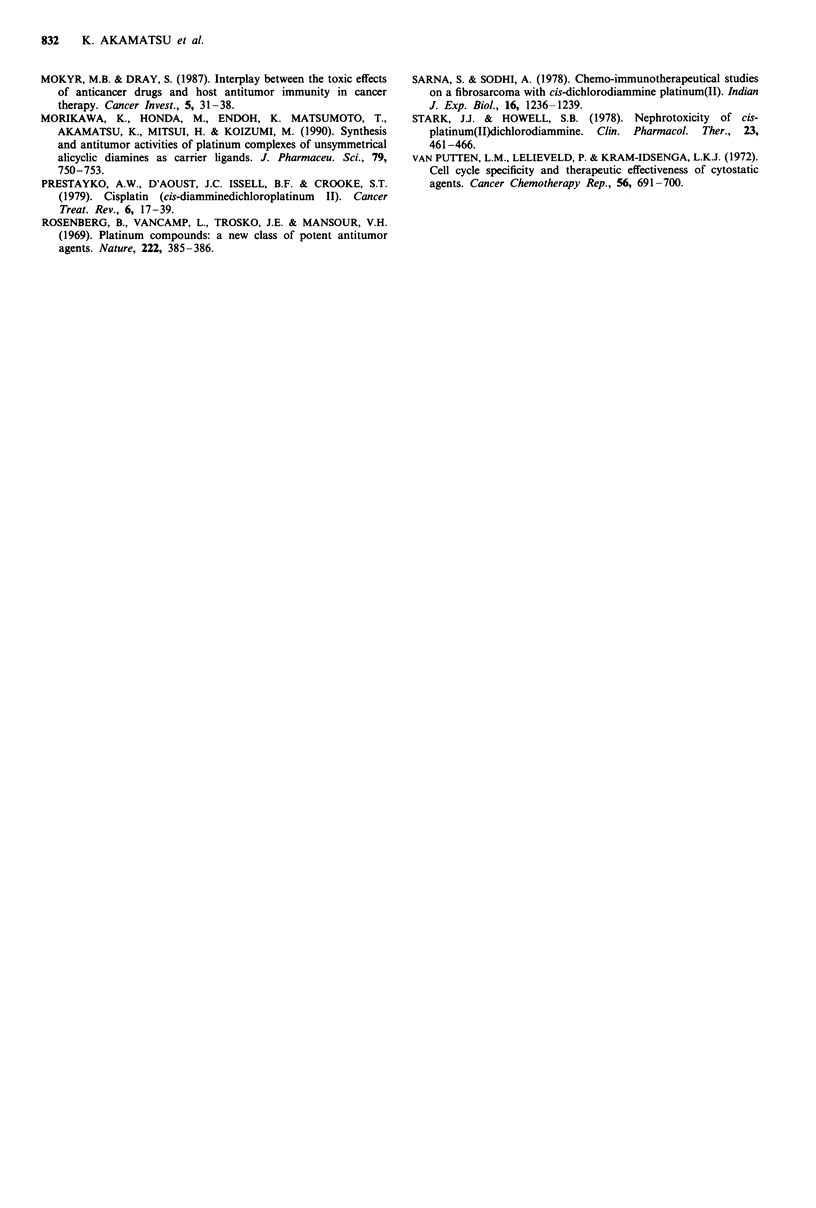# Potent antitumour activity of (-)-(R)-2-aminomethylpyrrolidine (1,1-cyclobutanedicarboxylato)platinum(II) monohydrate (DWA2114R) against advanced L1210 leukaemia in mice.

**DOI:** 10.1038/bjc.1992.368

**Published:** 1992-11

**Authors:** K. Akamatsu, K. Endo, T. Matsumoto, K. Kamisango, K. Morikawa, M. Koizumi, K. Koizumi

**Affiliations:** Exploratory Research Laboratories, Chugai Pharmaceutical Co. Ltd., Gotemba, Japan.

## Abstract

The time dependency of the antitumour activity of (-)-(R)-2-aminomethylpyrrolidine(1,1-cyclobutanedicarboxylato++ +)platinum(II) monohydrate (DWA2114R) was examined in mice inoculated i.p. with 10(5) mouse L1210 leukaemia cells. The increase in life span was greater in mice treated with 72 mg kg-1 DWA2114R on the 6th day following tumour inoculation than in mice treated at earlier times. Such superior effects against advanced L1210 were also seen with cis-diammine (1,1-cyclobutanedicarboxylato)platinum(II) (CBDCA) but not seen with the parent compound, cis-diamminedichloroplatinum(II) (CDDP) or other antitumour agents devoid of platinum. After the injection of DWA2114R on day 6, most of the ascites tumour cells accumulated in the S and G2/M phases of the cell cycle and the total cell number markedly decreased from 10(8) to less than 10(6). On the other hand, only a temporary G1 arrest and a less than 50% reduction of the cell number were induced after a similar treatment on day 3. Interestingly, the superiority of DWA2114R for advanced L1210 was lost in athymic nude mice and mice depleted of T cells by anti-thymocyte antisera. In addition, mice cured of advanced L1210 specifically rejected re-inoculated L1210 cells. These results indicate that the superior antitumour activity against advanced L1210 is unique to DWA2114R among the agents tested (except for CBDCA) and is caused by both an increased drug susceptibility of tumour cells and a drug-induced antitumour effect mediated by T cells of the host mice.


*Br. J. Cancer* (1992), **66**, 827–832 © Macmillan Press Ltd., 1992

### Potent antitumour activity of (–)-(R)-2-aminomethylpyrrolidine (1,1-cyclobutanedicarboxylato)platinum(II) monohydrate (DWA2114R) against advanced L1210 leukaemia in mice

K. Akamatsu[1], K. Endo, T. Matsumoto, K. Kamisango, K. Morikawa, M. Koizumi & K. Koizumi

*Exploratory Research Laboratories, Chugai Pharmaceutical Co. Ltd., Gotemba, Japan.*

**Summary** The time dependency of the antitumour activity of (–)-(R)-2-aminomethylpyrrolidine(1,1-cyclobutanedicarboxylato)platinum(II) monohydrate (DWA2114R) was examined in mice inoculated i.p. with $10^5$ mouse L1210 leukaemia cells. The increase in life span was greater in mice treated with 72 mg kg$^{-1}$ DWA2114R on the 6th day following tumour inoculation than in mice treated at earlier times. Such superior effects against advanced L1210 were also seen with *cis*-diammine (1,1-cyclobutanedicarboxylato)platinum(II) (CBDCA) but not seen with the parent compound, *cis*-diamminedichloroplatinum(II) (CDDP) or other antitumour agents devoid of platinum. After the injection of DWA2114R on day 6, most of the ascites tumour cells accumulated in the S and $G_2$/M phases of the cell cycle and the total cell number markedly decreased from $10^8$ to less than $10^6$. On the other hand, only a temporary $G_1$ arrest and a less than 50% reduction of the cell number were induced after a similar treatment on day 3. Interestingly, the superiority of DWA2114R for advanced L1210 was lost in athymic nude mice and mice depleted of T cells by anti-thymocyte antisera. In addition, mice cured of advanced L1210 specifically rejected re-inoculated L1210 cells. These results indicate that the superior antitumour activity against advanced L1210 is unique to DWA2114R among the agents tested (except for CBDCA) and is caused by both an increased drug susceptibility of tumour cells and a drug-induced antitumour effect mediated by T cells of the host mice.

The antitumour activity of *cis*-diamminedichloroplatinum(II) (CDDP) was first described more than 20 years ago (Rosenberg *et al.*, 1969). CDDP is one of the most potent antitumour drugs for a variety of human cancers in the clinic (Loehrer & Einhorn, 1984). However, the severe side effects of CDDP such as nephrotoxicity, nausea and vomiting, myelotoxicity, and ototoxicity are dose limiting factors for constraining clinical use (Stark & Howell, 1978; Prestayko *et al.*, 1979). A great effort has been made world wide to develop new platinum analogs with improved antitumour activity and reduced toxicity. In our laboratories, a series of platinum complexes carrying asymmetrical alicyclic diamines have been tested (Morikawa *et al.*, 1990). From these compounds, (–)-(R)-2-aminomethylpyrrolidine (1,1-cyclobutane-dicaboxylato)platinum(II) monohydrate (DWA2114R) has been selected as an agent with improved nephrotoxicity and potent antitumour activity (Matsumoto *et al.*, 1991).

We have revealed that single i.v. injection (day 1) of DWA2114R showed potent antitumour activity against L1210 implanted s.c. (Endo *et al.*, 1992). Thereafter, in preliminary experiments using advanced L1210 leukaemia in mice, DWA2114R showed an unexpectedly good therapeutic effect. This result made us very interested in the time dependency of the antitumour effect of DWA2114R, since antitumour agents are generally thought to be less active against tumours in advanced stages of progression. In this study, the time dependency of the antitumour activity of DWA2114R and its mechanisms were examined in mice inoculated with L1210 leukaemia cells. The results show that DWA2114R is more active against L1210 in advanced stages than in early stages. The analysis of the antitumour mechanisms in this model suggested that both the direct tumoricidal activity of the drug and T cell mediated immunity of the host mice play important roles in the superior effect of DWA2114R against advanced L1210.

Correspondence: K. Akamatsu, Fuji-Gotemba Research Labs., Chugai Pharmaceutical Co. Ltd., Komakado 1–135, Gotemba-shi, Shizuoka, 412, Japan.
Received 21 April 1992; and in revised form 19 June 1992.

### Materials and methods

### Drugs

DWA2114R and CBDCA were synthesised in our laboratories by the method described previously (Morikawa *et al.*, 1990) and CDDP was purchased from Aldrich Chemical Co. 5-fluorouracil and doxorubicin were purchased from Kyowa Hakkou Co. Ltd. (Tokyo), cyclophosphamide was from Shionogi Pharmaceutical Inc. (Osaka), etoposide was from Nihon Kayaku (Tokyo) and vincristine sulfate was from Sigma Chemical Co. (St Louis), respectively. Rabbit anti-mouse thymocyte, anti-asialo GM1 antisera and normal rabbit IgG were purchased from Wako Pure Chemical Industries Ltd. (Tokyo). All agents were dissolved in saline immediately before injection.

### Animals

Murine L1210 leukaemia cells were passaged in male 6-week-old DBA/2 mice once a week by intraperitoneal (i.p.) inoculation of $10^6$ cells and used to evaluate the drug effects in male 7-week-old (BALB/c × DBA/2)$F_1$ ($CDF_1$) mice. Both strains were supplied by Charles River, Japan. Testing was also done in male 6-week-old BALB/c nu/nu mice supplied by Japan Clea Inc. All mice were housed in an air conditioned room with a 12 h light–12 h dark cycle and given food (CE-2: Japan Clea Inc.) and tap water *ad libitum.*

### In vivo experimental procedures

L1210 cells were harvested from ascites fluid of tumour-bearing DBA/2 mice, and $10^5$ cells suspended in 0.2 ml of RPMI 1640 medium (GIBCO, Grand Island, NY) were i.p. inoculated into $CDF_1$ mice on day 0. All drugs were i.p. injected in a single dose on the appropriate day. Rabbit antisera were i.p. injected 4 h before the injection of DWA2114R on days 3 or 6. Survival of the mice was monitored and the median survival days of each group was determined. The injection doses of platinum complexes were determined on the basis of the lethal dose of each drug in mice. Three quarters of the $LD_{10}$ of each platinum complex was determined to be 72 mg $kg^{-1}$ for DWA2114R, 106 mg $kg^{-1}$ for CBDCA and 13 mg $kg^{-1}$ for CDDP. Statistical analysis of the antitumour activities of the drugs was conducted by the generalised Wilcoxon test and a *P* value less than 0.05 was defined as a statistically significant result. To evaluate the cytocidal activity of the drugs *in vivo*, the number and distribution of the cell cycle of ascites cells of tumour-bearing mice treated with drugs or vehicle were estimated at different times after tumour inoculation. The viability of cells harvested by peritoneal lavage with RPMI 1640 medium was determined by the trypan blue dye exclusion method.

### *Cell cycle analysis*

Ascites cells for flow cytometrical analysis were prepared by a procedure described in detail elsewhere (Akamatsu *et al.*, 1991). Briefly, cells were fixed with 70% ethanol immediately after the harvest. The fixed cells were treated with RNase to digest double-strand RNA, then stained with solution of 50 μg $ml^{-1}$ propidium iodide. The stained samples were analysed for DNA content with a FACScan (Becton Dickinson, CA).

### Results

### *Time dependency of the antitumour activities of DWA2114R and other antitumour agents*

The effect of the timing of drug-injection on the antitumour activities of DWA2114R was examined in mice i.p. inoculated with $10^5$ L1210 cells (Table I). The mice were injected with a single dose of 48 or 72 mg $kg^{-1}$ DWA2114R on days 1, 3, 6 or 8 after the inoculation of L1210 cells. Interestingly, DWA2114R showed its highest antitumour activity by injection on day 6 at both doses. The median survival time of the mice injected with 72 mg $kg^{-1}$ DWA2114R on day 6 was about 2-times greater than that on day 1 in both experiments. However, such an increase in survival time was lost by injection on day 8. Interestingly, a similar enhanced activity against advanced L1210 was also shown by equitoxic dose of CBDCA but not shown by CDDP (Table II). CBDCA as well as DWA2114R showed the greatest antitumour activity after the injection on day 6. On the other hand, the survival time of the mice treated with CDDP was longest in early injection (day 2) and decreased slightly in progressive order on days 4, 6, and 8.

The time dependency of antitumour activity was also examined for other types of antitumour agents which are devoid of platinum. Four mg $kg^{-1}$ doxorubicin, 60 or 80 mg $kg^{-1}$ 5-fluorouracil, 100 mg $kg^{-1}$ cyclophosphamide, 1 or 2 mg $kg^{-1}$ vincristine sulfate or 20 mg $kg^{-1}$ etoposide was i.p. injected into tumour-bearing mice in an early stage (day 2 or 3) or late stage (day 6) of tumour progression (Table III). These agents were more effective by early injection than late, although the differences were not statistically significant in the case of cyclophosphamide or etoposide. The enhancement of antitumour activity by late injection was shown only in DWA2114R among these agents.

### *Number of ascites tumour cells after DWA2114R injection*

In order to determine the effect of DWA2114R on tumour cell proliferation, the number of ascites tumour cells was monitored after inoculation of $10^5$ L1210 cells. As shown in Figure 1, the number of tumour cells per mouse in the control group increased by 10-fold ($10^6$/mouse) on day 3 and by 1000-fold ($10^8$/mouse) on day 6. Maximally, about $3 \times 10^8$ cells were present on day 7 and maintained this level until the animal's death. In the mice injected with 72 mg $kg^{-1}$ DWA2114R on day 3, the ascites cell number decreased by 50% on day 6 and maintained this level until day 9. However, the number then rapidly increased and reached about $10^8$ cells per mouse on day 13. In contrast, the $10^8$ ascites cells number was drastically decreased by more than 99% after the same treatment with the drug on day 6, and less than $10^6$ cells remained on day 15. These results are consistent with the therapeutic effect of DWA2114R mentioned above.

### *Cell cycle analysis*

The *in vivo* effect of DWA2114R on the cell cycle progression of ascites cells was analysed by using a flow cytometer (Figure 2). Most of the cells from mice without drug treatment on day 3 were present in the $G_1$ phase of the cell cycle. In contrast, the non-treated ascites cells on day 6 showed a typical cell cycle pattern of exponentially proliferating cells and the ratio of the cells in the S and $G_2$/M phases was much higher than that on day 3. The injection of DWA2114R on day 3 slightly decreased the cells in the S phase but increased the $G_1$ peak following 72 h (day 3 to 6). However, the ratio of cells in the S and $G_2$/M phases was markedly increased 240 h (day 13) after the drug injection, consistent with the increase in the cell number as shown in Figure 1. In contrast, the day 6-injection of DWA2114R drastically decreased the $G_1$ peak and most of the cells accumulated in S and $G_2$/M in the following 72 h (day 7–9). These cells in S and $G_2$/M then, almost disappeared and the remaining cells almost

**Table I** Survival time of $CDF_1$ mice injected with DWA2114R at various times after inoculation of $10^5$ L1210 cells

| *Treatment (mg $kg^{-1}$)* | *Day of drug injection* | *Survival days Median* | *Range* | *Long term survival*[a] | *None* | *P< vs*[b] | *Day 6* |
|---|---|---|---|---|---|---|---|
| Exp. 1 | | | | | | | |
| None | | 10.0 | 10–13 | 0/10 | | | |
| DWA2114R | 1 | 16.0 | 13–16 | 0/6 | 0.001 | 0.05 | |
| (48) | 3 | 17.0 | 16–19 | 0/5 | 0.001 | NS | |
| | 6 | 18.0 | 14–>40 | 1/5 | 0.001 | | |
| | 8 | 10.0 | 10 | 0/5 | 0.01 | 0.01 | |
| DWA2114R | 1 | 14.0 | 13–>40 | 1/6 | 0.01 | | 0.05 |
| (72) | 3 | 19.0 | 17–21 | 0/5 | 0.01 | | NS (0.1) |
| | 6 | 31.0 | 17–>40 | 2/5 | 0.01 | | |
| | 8 | 10.0 | 10–>40 | 1/5 | NS | | NS (0.1) |
| Exp. 2 | | | | | | | |
| None | | 9.0 | 9 | 0/6 | | | |
| DWA2114R | 1 | 16.5 | 14–19 | 0/6 | 0.01 | 0.01 | |
| (72) | 3 | 17.0 | 16–20 | 0/6 | 0.01 | 0.01 | |
| | 6 | 34.0 | 19–>40 | 3/6 | 0.01 | | |
| | 8 | 9.0 | 9 | 0/6 | NS | 0.01 | |

[a]Survival of the mice was observed for 40 days after inoculation of L1210 cells. [b]Statistical significance of survival time of the drug-treated groups *vs* that of the control (None) or that of the day 6-injected group (Day 6) at each dose was evaluated by generalised Wilcoxon test. NS = not significant.

**Table II** Comparison of time dependency in antitumour activities of platinum complexes against $CDF_1$ mice inoculated with $10^5$ L1210 cells

| *Treatment (mg kg$^{-1}$)* | *Day of drug injection* | *Survival days Median* | *Range* | *Long term survival* | *P< vs[b] None* | *Day 6* |
|---|---|---|---|---|---|---|
| None | | 10.0 | 9-12 | 0/10 | | |
| DWA2114R | 2 | 16.0 | 16-17 | 0/5 | 0.001 | 0.01 |
| (72) | 4 | 19.0 | 17-23 | 0/5 | 0.001 | 0.01 |
| | 6 | 28.0 | 24->30 | 2/5 | 0.001 | |
| | 8 | 9.5 | 9-10 | 0/6 | 0.05 | 0.01 |
| CBDCA | 2 | 18.0 | 16-18 | 0/5 | 0.01 | NS |
| (106) | 4 | 19.0 | 16-21 | 0/5 | 0.01 | NS |
| | 6 | 24.0 | 17->30 | 1/5 | 0.01 | |
| | 8 | 10.0 | 10->30 | 1/5 | NS | 0.05 |
| CDDP | 2 | 21.0 | 16->30 | 1/5 | 0.01 | NS |
| (13) | 4 | 21.0 | 19-24 | 0/5 | 0.01 | NS |
| | 6 | 19.0 | 18-22 | 0/6 | 0.01 | |
| | 8 | 9.0 | 9-15 | 0/6 | 0.05 | 0.05 |

[a]Survival of the mice was observed for 30 days after inoculation of L1210 cells. [b]Statistical significance of survival time of the drug-treated group *vs* that of the control (None) or that of the day 6-injected group (Day 6) in each drug was evaluated by generalised Wilcoxon test. NS = not significant.

**Table III** Comparison between survival time of $CDF_1$ mice injected with the antitumour drug on day 2 or day 3 and that on day 6 after inoculation of $10^5$ L1210 cells

| *Treatment (mg kg$^{-1}$)* | *Day of drug injection* | *Survival days Median* | *Range* | *Long term survival* | *P<[b]* |
|---|---|---|---|---|---|
| Exp. 1 | | | | | |
| Saline | 2 | 9.0 | 9-12 | 0/7 | |
| | 6 | 9.0 | 9-11 | 0/7 | NS |
| Doxorubicin | 2 | 18.5 | 15-23 | 0/6 | |
| (4) | 6 | 10.5 | 10-12 | 0/6 | 0.01 |
| 5-fluorouracil | 2 | 14.0 | 13-15 | 0/6 | |
| (60) | 6 | 12.0 | 11-12 | 0/6 | 0.01 |
| Cyclophosphamide | 2 | 18.5 | 17->40 | 1/6 | |
| (100) | 6 | 18.5 | 15-21 | 0/6 | NS |
| Vincristine sulfate | 2 | 13.0 | 11-14 | 0/6 | |
| (1) | 6 | 10.0 | 10 | 0/6 | 0.01 |
| Exp. 2 | | | | | |
| Saline | 3 | 9.0 | 9-10 | 0/6 | |
| | 6 | 9.0 | 9-11 | 0/6 | NS |
| 5-fluorouracil | 3 | 17.0 | 15-18 | 0/6 | |
| (80) | 6 | 13.0 | 12-13 | 0/6 | 0.01 |
| Cyclophosphamide | 3 | 20.5 | 19-29 | 0/6 | |
| (100) | 6 | 19.0 | 19-20 | 0/6 | NS |
| Vincristine sulfate | 3 | 14.5 | 12-18 | 0/6 | |
| (2) | 6 | 12.0 | 11-13 | 0/6 | 0.05 |
| Etoposide | 3 | 14.0 | 11-16 | 0/5 | |
| (20) | 6 | 12.0 | 11-12 | 0/5 | NS |
| DWA2114R | 3 | 20.5 | 19->40 | 1/6 | |
| (72) | 6 | 40.0 | 31->40 | 5/6 | 0.05 |

[a]Survival of the mice was observed for 40 days after inoculation of L1210 cells. [b]Generalised Wilcoxon test was used for the statistical evaluation. NS = not significant.

appeared in the $G_1$ phase 168 h after drug injection (day 13). At this time, about $2 \times 10^6$ tumour cells were present in the peritoneal cavity, as described in Figure 1.

### *The role of the host immune system in survival of drug-treated mice*

We checked whether T cells of the host mice contributed to the superior effect of DWA2114R against advanced L1210 since T cells are thought to be important for the elimination of tumour cells in advanced stages of tumour progression. Seventy two or 48 mg kg$^{-1}$ DWA2114R was i.p. injected into athymic BALB/c nude mice on days 1, 3, 6 or 8 after i.p. inoculation with $10^5$ L1210 cells. As shown in Table IV, the median survival time of the control mice was 9 days, which was almost the same as that of $CDF_1$ mice as described above (Tables I, II and III). However, no advantage of injection in advanced stage of tumour progression on the antitumour activity of DWA2114R was seen in the nude mice. DWA2114R showed its highest activity after the injection on day 1 at both doses and the activity decreased in the order: days 3, 6 and 8.

A critical role of T cells was also convincingly demonstrated using anti-thymocyte antisera. $CDF_1$ mice inoculated with L1210 cells were treated with rabbit anti-thymocyte or anti-asialo GM1 antisera to eliminate T cells (Kataoka *et al.*, 1985) or NK cells (Habu *et al.*, 1981) 4 h before the drug injection. As shown in Figure 3, pretreatment with anti-thymocyte antisera reduced the antitumour activity of DWA2114R injected on day 3 (about 30% reduction: not significant) or on day 6 (about 50% reduction: $P<0.05$). On the other hand, the injection of anti-asialo GM1 had no effect on the activity of DWA2114R.

We then examined whether the mice cured of L1210

**Figure 1** Change of the number of ascites L1210 cells after i.p. injection of 72 mg $kg^{-1}$ DWA2114R. $CDF_1$ mice were i.p. inoculated with $10^5$ L1210 cells on day 0 and given DWA2114R on day 3 (▲) or day 6 (■). Closed circles represent controls. Points indicate mean number of three or four mice and bars indicate s.d.

**Figure 2** Effects of i.p. injection of 72 mg $kg^{-1}$ DWA2114R on the cell cycle progression of ascites L1210 cells. $CDF_1$ mice were i.p. inoculated with $10^5$ L1210 cells on day 0 and given DWA2114R on day 3 or day 6. Cells were harvested from the peritoneal cavity at the time indicated in the figure and analysed using flow cytometer.

leukaemia by treatment with DWA2114R could eliminate reinoculated L1210 cells. Six mice survived for 35 days after 50 $CDF_1$ mice inoculated with $10^5$ L1210 cells per mouse were injected with 72 mg $kg^{-1}$ DWA2114R on day 6. Three of these cured mice were reinoculated with $10^5$ L1210 cells, the remaining 3 mice with $10^5$ P388 leukaemia cells. All the mice reinoculated with L1210 cells survived for more than 60 days, whereas none of them inoculated with P388 cells survived more than 20 days.

### Discussion

In this study, the antitumour activity of DWA2114R against L1210 leukaemia was shown to be significantly higher in late than in early stage of the disease. The survival time of the mice injected with 72 mg $kg^{-1}$ DWA2114R on day 6 was about 2-times greater than that on day 1 (Table I). Such time dependency of the antitumour activity is unique to two CDDP analogs, DWA2114R and CBDCA since the parent compound CDDP and other antitumour agents showed higher antitumour activity in the early rather than in the late administration by injection (Tables II and III). These results suggest that DWA2114R and CBDCA have certain unique properties in their antitumour mechanisms.

The kinetics of the proliferation and the cell cycle progression of the ascites cells suggest that the susceptibility of the L1210 cells to DWA2114R is much higher on day 6 than on day 3 (Figures 1 and 2). The injection of DWA2114R on day 6 induced S and $G_2$/M arrest, whereas the same treatment on day 3 only slightly induced $G_1$ arrest. From the results of our experiments using exponential L1210 cells in culture, we previously reported that $G_2$ arrest is critical for the cytotoxicity of DWA2114R, whereas $G_1$ arrest was not observed under such conditions (Akamatsu *et al.*, 1991). This may explain why most of exponential cells on day 6 are highly susceptible to the cytotoxicity of DWA2114R. On the other

**Table IV** Survival time of BALB/c athymic nude mice injected with DWA2114R at various times after inoculation with $10^5$ L1210 cells

| *Treatment (mg $kg^{-1}$)* | *Day of drug injection* | *Survival days Median* | *Range* | *Long term survival*[a] | *P< vs*[b] *None* | *Day 6* |
|---|---|---|---|---|---|---|
| None | | 9.0 | 9–11 | 0/6 | | |
| DWA2114R | 1 | 15.5 | 13–23 | 0/6 | 0.01 | 0.05 |
| (48) | 3 | 15.0 | 14–16 | 0/6 | 0.01 | 0.01 |
| | 6 | 13.5 | 12–14 | 0/6 | 0.01 | |
| | 8 | 9.0 | 9–11 | 0/6 | NS | 0.01 |
| DWA2114R | 1 | 19.0 | 15–>40 | 1/6 | 0.01 | 0.01 |
| (72) | 3 | 16.0 | 15–17 | 0/6 | 0.01 | 0.01 |
| | 6 | 14.0 | 14–15 | 0/6 | 0.01 | |
| | 8 | 9.0 | 9–10 | 0/6 | NS | 0.01 |

[a]Survival of the mice was observed for 40 days after inoculation of L1210 cells. [b]Statistical significance of survival time of the drug-treated group *vs* that of the control (None) or that of the day 6- injected group (Day 6) at each dose was evaluated by generalised Wilcoxon test. NS = not significant.

**Figure 3** Reduction of the antitumour activity of DWA2114R by anti-thymocyte antisera. $CDF_1$ mice inoculated with $10^5$ L1210 cells were i.p. injected with anti-thymocyte antisera (Thy), anti-asialo GM1 antisera (AsGM1) or normal rabbit IgG (NRG) 4 h before the injection of 72 mg $kg^{-1}$ DWA2114R on day 3 (open column) or day 6 (dotted column). Columns indicate mean survival days of mice and bars indicate s.e.

hand, the resting cells, which are the majority of cells on day 3, may be arrested in $G_1$ in a reversible fashion. A direct comparison of the effect of DWA2114R on exponential cells with that on resting cells will be required to confirm this hypothesis.

However, the enhanced drug susceptibility in exponential cells is not unique to DWA2114R. Almost all antitumour agents are more active against exponentially growing cells as compared to resting ones (Bruce *et al.*, 1966; Van Putten *et al.*, 1972; Bhuyan *et al.*, 1977). In addition, the flow cytometrical pattern of the drug-treated cells after the day 6-injection of DWA2114R showed no increase in cell debris. This means a large proportion of the cells arrested in S and $G_2/M$ must be rapidly eliminated from the peritoneal cavity of host mice. This suggests that the superiority of DWA2114R against advanced L1210 should be a result of certain unique actions mediated by the immune systems of host mice. In this regard, the contribution of T cells to the antitumour activity of DWA2114R was reasoned as follows. The superiority of DWA2114R against advanced L1210 was not observed in athymic nude mice (Table IV) and was lost in mice depleted of T cells by injection of anti-thymocyte antisera (Figure 3). The reduction in the antitumour activity of DWA2114R by the injection of such antisera was larger in the mice treated on day 6 than in those treated on day 3. Moreover, mice cured of advanced L1210 rejected reinoculated L1210 cells. These results suggest that T cells are essential for the superior effect of DWA2114R against advanced tumour and contribute to the establishment of tumour specific immunity.

Although the experimental systems were different, the immuno-potentiating activity against tumour cells has also been seen with other antitumour agents (Kleinerman & Zwelling, 1982; Mokyr & Dray, 1987). It has also been reported that antitumour immunity is induced in mice treated with CDDP (Kociba *et al.*, 1970; Sarna & Sodhi, 1978). In an *in vitro* system, CDDP has been reported to activate spontaneous cell-mediated cytotoxicity (Kleinerman *et al.*, 1980; Mally *et al.*, 1980) but not to activate T cell mediated cytotoxicity (Mally *et al.*, 1979). These reports indicate that CDDP may have some ability to augment host antitumour immunity. However, the specificity of these rejection responses to tumour and the uniqueness of CDDP to induce these rejections were not examined. It is fascinating to consider the possibility that DWA2114R has unique ability to take advantage of T cell-mediated antitumour immunity. In this regard, it is suggestive that CBDCA which has the same slow-reacting leaving groups as DWA2114R showed a similar time dependency in its antitumour activity. Such similarity between DWA2114R and CBDCA raises the possibility that slow-reacting leaving groups might cause the differences observed between CDDP and these two compounds. More precise analysis of the role of the immune systems in the host mice using various CDDP analogs would elucidate the critical difference of these compounds.

In conclusion, at least two mechanisms are involved in the superior effect of DWA2114R against advanced L1210 leukaemia. First, an increased drug-sensitivity of the tumour cells. Second, T cell-mediated antitumour immunity in the host mice. As discussed above, the latter is thought to be critical for the unique effect of DWA2114R in this model. This experimental system should be useful to elucidate the role of immunological function in the therapeutic effect of the antitumour platinum complexes.

### References

AKAMATSU, K., ENDO, K., MATSUMOTO, T., MORIKAWA, K., KOIZUMI, M., MITSUI, H. & KOIZUMI, K. (1991). *In vitro* antitumor mechanism of a new platinum complex, (−)-(R)-2-aminomethylpyrrolidine (1,1-cyclobutanedicarboxylato) platinum (II). *Anticancer Res.*, **11**, 151–156.

BHUYAN, B.K., FRASER, T.J. & DAY, K.J. (1977). Cell proliferation kinetics and drug sensitivity of exponential and stationary populations of cultured L1210 cells. *Cancer Res.*, **37**, 1057–1063.

BRUCE, W.R., MEEKER, B.E. & VALERIOTTE, F.A. (1966). A comparison of the sensitivity of normal hematopoietic and transplanted lymphoma colony-forming cells to chemotherapeutic agents administered *in vivo*. *J. Natl Cancer Inst.*, **37**, 233–245.

ENDO, K., AKAMATSU, K., MATSUMOTO, T., MORIKAWA, K., KOIZUMI, M., MITSUI, H. & KOIZUMI, K. (1992). Antitumor effects of three platinum complexes, (−)-(R)-2-aminomethylpyrrolidine (1,1-cyclobutanedicarboxylato) platinum (II) monohydrate (DWA2114R), *cis*-diammine(1,1-cyclobutanedicarboxylato) platinum(II) (CBDCA) and *cis*-diamminedichloroplatinum(II) (CDDP), in mice. *Anticancer Res.*, **12**, 49–58.

HABU, S., FUKUI, H., SHIMAMURA, K., OKUMURA, K. & TAMAOKI, N. (1981). *In vivo* effects of anti-asialo GM1. I. Reduction of NK activity and enhancement of transplanted tumour growth in nude mice. *J. Immunol.*, **127**, 34–38.

KATAOKA, T., MATSUURA, N., OH-HASHI, F. & SUHARA, Y. (1985). Treatment regimen and host T-cell-dependent therapeutic effect of interferon in mouse solid tumors. *Cancer Res.*, **45**, 3548–3553.

KLEINERMAN, E.S., ZWELLING, L.A. & MUCHMORE, A.V. (1980). The enhancement of naturally occurring spontaneous monocyte mediated cytotoxicity by *cis*-diamminedichloroplatinum(II). *Cancer Res.*, **40**, 3099–3102.

KLEINERMAN, E.S. & ZWELLING, L.A. (1982). The effect of *cis*-diamminedichloroplatinum(II) on immune function *in vitro* and *in vivo*. *Cancer Immunol. Immunother.*, **12**, 191–196.

KOCIBA, R.J., SLEIGHT, S.D. & ROSENBERG, B. (1970). Inhibition of Dunning ascitic leukemia and Walker 256 carcinoma with *cis*-diamminedichloroplatinum (NSC-119874). *Cancer Chemother. Rep.*, **54**, 325–328.

LOEHRER, P.J. & EINHORN, L.H. (1984). *Cisplatin. Ann. Intern. Med.*, **100**, 704–713.

MALLY, M.B., TAYLOR, R.C. & CALLEWAERT, D.M. (1979). Effect of platinum antitumor agents on *in vitro* assays of human antitumor immunity. I. Effect of *cis*-$[Pt(NH_3)_2Cl_2]$ on the mixed lymphocyte tumor assay. *Chemotherapy*, **25**, 117–128.

MALLY, M.B., TAYLOR, R.C. & CALLEWAERT, D.M. (1980). Effect of platinum antitumor agents on *in vitro* assays of human antitumor immunity. II. Effect of *cis*-$[Pt(NH_3)_2Cl_2]$ on spontaneous cell-mediated cytotoxicity. *Chemotherapy*, **26**, 1–6.

MATSUMOTO, T., ENDOH, K., AKAMATSU, K. KAMISANGO, K., MITSUI, H., KOIZUMI, K., MORIKAWA, K., KOIZUMI, M. & MATSUNO, T. (1991). Comparison of the antitumor effects and nephrotoxicity-inducing activities of two new platinum complexes, (−)-(R)-2-aminomethylpyrrolidine(1,1-cyclobutanedicarboxylato)platinum(II) monohydrate, and its enantiomeric isomer. *Br. J. Cancer.*, **64**, 41–46.

MOKYR, M.B. & DRAY, S. (1987). Interplay between the toxic effects of anticancer drugs and host antitumor immunity in cancer therapy. *Cancer Invest.*, **5**, 31–38.

MORIKAWA, K., HONDA, M., ENDOH, K. MATSUMOTO, T., AKAMATSU, K., MITSUI, H. & KOIZUMI, M. (1990). Synthesis and antitumor activities of platinum complexes of unsymmetrical alicyclic diamines as carrier ligands. *J. Pharmaceu. Sci.*, **79**, 750–753.

PRESTAYKO, A.W., D'AOUST, J.C. ISSELL, B.F. & CROOKE, S.T. (1979). Cisplatin (*cis*-diamminedichloroplatinum II). *Cancer Treat. Rev.*, **6**, 17–39.

ROSENBERG, B., VANCAMP, L., TROSKO, J.E. & MANSOUR, V.H. (1969). Platinum compounds: a new class of potent antitumor agents. *Nature*, **222**, 385–386.

SARNA, S. & SODHI, A. (1978). Chemo-immunotherapeutical studies on a fibrosarcoma with *cis*-dichlorodiammine platinum(II). *Indian J. Exp. Biol.*, **16**, 1236–1239.

STARK, J.J. & HOWELL, S.B. (1978). Nephrotoxicity of *cis*-platinum(II)dichlorodiammine. *Clin. Pharmacol. Ther.*, **23**, 461–466.

VAN PUTTEN, L.M., LELIEVELD, P. & KRAM-IDSENGA, L.K.J. (1972). Cell cycle specificity and therapeutic effectiveness of cytostatic agents. *Cancer Chemotherapy Rep.*, **56**, 691–700.